# More sedentary behavior and lower physical activity levels in pregnant women during the COVID-19 pandemic

**DOI:** 10.1038/s41598-025-24511-9

**Published:** 2025-10-20

**Authors:** Emelie Lindberger, Fredrik Ahlsson, Henrik Johansson, Inger Sundström Poromaa, Anna-Karin Wikström

**Affiliations:** Department of Women’s and Children’s Health, Uppsala University, 751 85 Uppsala, Sweden

**Keywords:** Pregnancy, COVID-19, Sedentary behavior, Physical activity, Diseases, Health care, Medical research, Risk factors

## Abstract

This Swedish cohort study performed 2016‒2022 aimed to evaluate activity patterns before and during the COVID-19 pandemic among 1405 pregnant women. Sedentary behavior and physical activity levels were objectively measured during seven consecutive days by an accelerometer in early to mid-pregnancy. Linear regression models adjusted for age, parity, BMI, smoking, country of birth, and timing of measurements were used. A subgroup analysis was performed to evaluate whether activity patterns returned to pre-pandemic levels during year 2022. Compared with before COVID-19, daily sedentary behavior increased by 6.6% points (β 6.6, CI 5.4, 7.8), and daily physical activity levels decreased by 6.7% points (β − 6.7, CI − 7.8, − 5.7) during the pandemic. In 2022, daily sedentary behavior was still increased by 7.2% points (β 7.2, CI 5.3, 9.2), and daily physical activity levels decreased by 7.3% points (β − 7.3, CI − 9.1, − 5.5), compared with before COVID-19. Hence, there was a modest increase in sedentary behavior and a small decrease in physical activity after the pandemic outbreak, and these changes were still present during 2022. It is important that health providers are aware of the potential negative changes in activity patterns among pregnant women following a pandemic or a similar situation.

## Introduction

Physical activity is beneficial for most pregnant women. The American College of Obstetricians and Gynecologists (ACOG) recommends women with uncomplicated pregnancies to engage in at least 20–30 min of daily aerobic and strength-conditioning exercises, in line with the recommendations of the World Health Organization (WHO)^1,2^. During pregnancy, sedentary behavior (defined as low-intensity behavior while lying, reclining or sitting^3^) has been linked to gestational diabetes mellitus^4^, perinatal depression^4^, and preterm birth^5^. Contrarily, physical activity has been associated with less gestational weight gain^6^, lower risk of gestational diabetes mellitus^7^, lower risk of preeclampsia^8^, and reduced likelihood of infant admission to neonatal intensive care unit^5^. Reduced insulin resistance and improved uteroplacental blood flow has been suggested as possible mechanisms behind these associations^9^.

Although physical activity seems clearly beneficial for pregnancy outcomes, the field requires studies that utilize objective methods to assess activity patterns. The use of subjective methods, such as questionnaires or interviews, might be prone to bias as sedentary behavior tend to be underestimated and physical activity overestimated^10^. Hence, more objective pregnancy activity data are needed.

On 11 March 2020, the coronavirus disease 2019 (COVID-19) was declared to be a global pandemic by the WHO^11^. To limit disease transmission, governments advocated for social distancing and restricted population movement. The country-specific public health interventions differed in extent and timing, and the Swedish restrictive policies were less invasive compared with many other countries in Europe^12^.

After the outbreak of the pandemic, activity patterns changed across various populations. Two systematic reviews and meta-analyses report increased sedentary behavior and decreased physical activity during the first months after the pandemic outbreak^13,14^. Few studies have evaluated pregnant women´s sedentary behavior and physical activity in relation to the COVID-19 pandemic, and the results indicate that also pregnant women spent more time being sedentary and less time being physically active after the pandemic outbreak^15–18^. However, as the majority of these studies use retrospective and self-reported data, the results might be biased. In addition, further research is required to understand whether the alterations in activity patterns are lasting changes.

This study aimed to evaluate objectively measured early to mid-pregnancy sedentary behavior and physical activity levels before and during the COVID-19 pandemic in a large population-based cohort. To investigate whether changes in activity patterns persisted (at least short-term), a subgroup analysis comparing activity measurements from before COVID-19 with activity measurements from year 2022 was performed.

## Materials and methods

### Study design and participants

This cohort study was conducted at Uppsala University hospital, Uppsala, Sweden between 2016 and 2022. During the study period, 1713 pregnant women were recruited to the study in conjunction with the first or early second trimester ultrasounds, both offered all pregnant women. Eligible women had to be ≥ 18 years old, Swedish speaking, having an uncomplicated pregnancy at recruitment, and being able to wear a gadget, in the form of a wrist watch, during work hours. Study participation involved carrying an accelerometer for seven consecutive days for collection of sedentary behavior and physical activity levels. We obtained written informed consent from all participants at inclusion. The Regional Ethical Review Board in Uppsala approved this study 20 April 2016 (Dnr: 2016/142). All research was performed in accordance with relevant national and international guidelines.

### Swedish guidelines during COVID-19

Sweden had a less invasive response to COVID-19 compared with many other countries. There was no general lockdown; instead, the Swedish COVID-19 strategy aimed to slow the spread of the pandemic. Social distancing was strongly recommended and mandatory during public events, and in bars and restaurants. It was not allowed to visit nursing facilities. Facemasks were not compulsory outside of healthcare environments. Those who could work from home were urged to do so, and schools were open for children up to 16 years of age^19^.

### Data collection

The woman’s weight was measured at the first antenatal visit. Body mass index (BMI) was calculated as the weight in kilograms divided by the square of the height in meters. At inclusion, the woman filled in a questionnaire regarding information on maternal characteristics.

### Sedentary behavior and physical activity measurement

Sedentary behavior and physical activity levels were measured by the use of an accelerometer, either Actiwatch 2 or Actiwatch Spectrum Plus (Philips Respironics, Eindhoven, Netherlands). The Actiwatch monitor is worn as a wristwatch and registers anteroposterior, mediolateral, and vertical movements. The study participants were instructed to wear the accelerometer on their non-dominant wrist without interruption during all waking time for seven consecutive days, despite during water-based activities. They were instructed to live their daily lives as usual. A minimum of four valid days of physical activity recordings were required with ≥ 10 h of recordings/day. Movements were collected in 30-second epochs and reported as counts per minute (cpm)^20^ after processing by the use of Philips Actiware software. Physical activity was classified in four categories based on the cpm: sedentary, light, moderate, and vigorous, and time spent in each category was recorded^20^. We defined the physical intensity categories by the following cut-off points: sedentary activity: ≤256 cpm, moderate activity: 418–720 cpm, and vigorous activity: ≥721 cpm, as per Kemp et al.^21^ Due to low sensitivity and specificity of the accelerometer to characterize light activity (257–417 cpm)^21^, it was left out from further analyses. Hence, all study participants contributed with their proportion of time spent in sedentary behavior, moderate activity, and vigorous activity.

### Outcomes

Outcomes were differences in the proportion of time spent in sedentary behavior and the proportion of time spent in physical activity (the sum of the proportion of time spent in moderate and vigorous activity) between pregnant women who performed activity measurements prior to the COVID-19 pandemic outbreak, which was set at 11 March 2020 (the date the WHO declared COVID-19 a global pandemic) (“before COVID-19”, *n* = 939) and those who performed the measurements after (“during COVID-19”, *n* = 466). In addition, we evaluated difference in these activity measures between pregnant women performing measurements prior to the COVID-19 pandemic outbreak and those who performed the measurements during 2022 (“during late COVID-19”, *n* = 117).

### Statistical methods

IBM SPSS Statistics version 28 was used for statistical analyses. We considered a nominal two-sided *p*-value < 0.05 to indicate statistical significance.

Differences in mean time spent in sedentary behavior and physical activity between groups were evaluated by t-tests. Further, proportion of time spent in sedentary behavior and proportion of time spent in physical activity were compared between groups using linear regression models. Adjusted models were performed for each outcome including maternal age, parity, early pregnancy BMI, smoking habits, country of birth, and timing of activity measurements (at the first or early second trimester ultrasound examination).

To examine whether activity patterns resumed in the late pandemic, a subgroup analysis was performed comparing sedentary behavior and physical activity of the group “before COVID-19” with the subgroup “during late COVID-19”.

The results are presented as B coefficients (β) and 95% confidence interval (CI).

## Results

### Study population

Of the 1713 women enrolled to this study, 308 participants were excluded from further analyses due to the following: technical problems with the Actiware software (*n* = 56), insufficient wear time (*n* = 85), lost to follow-up (*n* = 125), multiple pregnancy (*n* = 18), abortion or miscarriage (*n* = 12), and certain data missing from pregnancy (*n* = 12). Hence, the final study population consisted of 1405 pregnant women.

Demographic characteristics of the before COVID-19, during COVID-19, and during late COVID-19 groups are presented in Table 1.

**Table 1:** Study population characteristics divided by group affiliation based on activity measurement period.

Variable	“Before COVID-19”-group *n* = 939	“During COVID-19”-group *n* = 466	“During late COVID-19”-group *n* = 117
Age, years (mean ± SD)^a^	31.4 ± 4.6	31.7 ± 4.0	32.0 ± 3.8
Birth country within the European region, n (%)	837 (89.1)	432 (92.7)	111 (94.9)
Nulliparous, n (%)^b^	443 (47.2)	248 (53.2)	71 (60.7)
Current smoker at first antenatal visit, n (%)^b^	86 (9.2)	25 (5.4)	2 (1.7)
Weight at first antenatal visit, kg (mean ± SD)^c^	69.6 ± 14.4	69.8 ± 13.4	71.3 ± 12.7
BMI at first antenatal visit, kg/m^2^ (mean ± SD)^d^	24.9 ± 4.8	25.1 ± 4.5	25.4 ± 4.2
Proportion of time spent in sedentary behavior, % (mean ± SD)	57.0 ± 10.2	65.0 ± 9.2	66.2 ± 8.6
Proportion of time spent in moderate activity, % (mean ± SD)	16.8 ± 4.5	13.3 ± 3.9	12.9 ± 3.7
Proportion of time spent in vigorous activity, % (mean ± SD)	11.8 ± 6.6	7.5 ± 3.9	6.9 ± 3.5
Sedentary behavior, hours per day (mean ± SD)	9.0 ± 1.6	10.0 ± 1.5	10.2 ± 1.4
Moderate activity, hours per day (mean ± SD)	2.7 ± 0.8	2.1 ± 0.6	2.0 ± 0.6
Vigorous activity, hours per day (mean ± SD)	1.9 ± 1.1	1.2 ± 0.6	1.1 ± 0.6

^a^Data missing in 1 individual.

^b^Data missing in 5 individuals.

^c^Data missing in 9 individuals.

^d^Data missing in 10 individuals.

SD, standard deviation; BMI, body mass index.

The measurements of sedentary behavior and physical activity levels were conducted between gestational weeks 8‒23. About two thirds of the study cohort (*n* = 966) were enrolled in conjunction with the first trimester ultrasound (median gestational length at start of measurements 90 days, interquartile range (IQR) 5 days). The remaining study cohort (*n* = 428) was enrolled in conjunction with the early second trimester ultrasound (median gestational length at start of measurements 133 days, IQR 7 days).

### Daily sedentary behavior and physical activity patterns

The measuring period ranged 4‒7 days (mean 6.7 days) and the mean daily waking wear time was 15.7 h (SD 1.1 h). The mean time spent in sedentary behavior was 9.0 h (SD 1.6) in the before COVID-19 group, 10.0 h (SD 1.5) in the during COVID-19 group, and 10.2 h (SD 1.4) in the during late COVID-19 group. Corresponding figures for physical activity were 4.6 h (SD 1.6), 3.2 h (SD 1.2), and 3.1 h (SD 1.1) respectively. The mean difference in sedentary time between the before COVID-19 group and the during COVID-19 group was − 0.9 h (CI − 1.1 to − 0.7, *p* < 0.001), and the mean difference in time being physical activity was 1.4 h (CI 1.2 to 1.5, *p* < 0.001). The distribution of daily sedentary behavior and physical activity levels across groups is presented in Table 1.

### Sedentary behavior and physical activity levels before and during COVID-19

In the crude analysis, daily sedentary behavior was 8.0% points higher in the during COVID-19-group compared with the before COVID-19-group (β 8.0, 95% CI 6.9 to 9.1, *p* < 0.001) (Table 2). The result was similar after adjustments for maternal age, parity, early pregnancy BMI, current smoking, country of birth, and timing of activity measurements, showing a 6.6% points increase in daily sedentary behavior (β 6.6, CI 5.4 to 7.8, *p* < 0.001) (Table 2). Furthermore, in the crude analysis, daily physical activity levels were 7.7% points lower in the during COVID-19-group compared with the before COVID-19-group (β − 7.7, CI − 8.7 to − 6.7, *p* < 0.001). After adjustments, the difference was 6.7% points (β − 6.7, CI − 7.8 to − 5.7, *p* < 0.001) (Table 2).

**Table 2:** Results from linear regression analyses comparing before and during COVID-19 pandemic sedentary behavior and physical activity levels.

	Unadjusted model			Adjusted model^a^		
	β	CI	*P*	β	CI	*P*
Proportion of time spent in sedentary behavior, %	8.0	6.9 to 9.1	< 0.001	6.6	5.4 to 7.8	< 0.001
Proportion of time spent in physical activity, %	− 7.7	− 8.7 to − 6.7	< 0.001	-6.7	− 7.8 to − 5.7	< 0.001

Data are B coefficients (β) and 95% confidence interval (CI).

Data were analyzed using linear regression models. The before COVID-19 group is the reference.

^a^Adjustments were made for maternal age, parity, early pregnancy BMI, smoking habits, country of birth, and timing of activity measurements.

### Subgroup analysis of before COVID-19 and during late COVID-19

The crude analysis showed an increase in daily sedentary behavior by 9.1% points in the during late COVID-19-group compared with the before COVID-19-group (β 9.1, CI 7.2 to 11.1, *p* < 0.001). After adjustments, the difference was 7.2% points (β 7.2, CI 5.3 to 9.2, *p* < 0.001) (Table 3). In the crude analysis, the during late COVID-19-group had 8.7% points lower daily physical activity levels compared with the before COVID-19-group (β − 8.7, CI − 10.5 to − 7.0, *p* < 0.001). In the adjusted analysis, the difference was 7.3% points (β − 7.3, CI − 9.1 to − 5.5, *p* < 0.001) (Table 3).

**Table 3:** Results from subgroup analyses comparing before and during late COVID-19 pandemic sedentary behavior and physical activity levels.

Outcome	Unadjusted model			Adjusted model^a^		
	β	CI	*P*	β	CI	*P*
Proportion of time spent in sedentary behavior, %	9.1	7.2 to 11.1	< 0.001	7.2	5.3 to 9.2	< 0.001
Proportion of time spent in physical activity, %	-8.7	− 10.5 to − 7.0	< 0.001	− 7.3	− 9.1 to − 5.5	< 0.001

Data are B coefficients (β) and 95% confidence interval (CI).

Data were analyzed using linear regression models. The before COVID-19 group is the reference.

^a^Adjustments were made for maternal age, parity, early pregnancy BMI, smoking habits, country of birth, and timing of activity measurements.

## Discussion

The results of this study showed a modest increase in sedentary behavior as well as a small decrease in physical activity levels among pregnant women in Sweden after the outbreak of the COVID-19 pandemic, and this change in activity patterns remained in 2022.

Our findings are in line with previous research. According to a US based study evaluating objectively assessed physical activity measurements in 228 pregnant women either during 2017‒2019 or during 2021‒2023, sedentary time increased and physical activity levels decreased after the COVID-19 pandemic outbreak^22^. Specifically, women who participated 2021‒2023 had an increase in daily sedentary time by 0.77–1.13 h, and a reduction in weekly physical activity levels by 57–77 min compared with women who participated 2017‒2019. A similar change in physical activity patterns before and after the pandemic outbreak has also been observed by others in the general population^13,14^.

We observed a modest increase in daily sedentary behavior by 0.9 h following the onset of the pandemic. Sedentary behavior during pregnancy has been linked to gestational diabetes mellitus and both prepartum and postpartum depression. However, the potential impact of this relatively modest change among our study participants on pregnancy outcomes remains uncertain.

In addition, our results showed a decrease in daily physical activity levels by 1.4 h during COVID-19. Physical activity during pregnancy has positive effects on the pregnant woman and the fetus^1^, for example less gestational weight gain^6^, lower risk of gestational diabetes mellitus^7^, and lower risk of preeclampsia and preterm delivery^8,23,24^. However, a Swedish registry study examining the risk of preterm birth and stillbirth after the pandemic outbreak with births between 2015 and 2019 reports no differences in the odds of these outcomes^25^. Hence, it is uncertain whether the reduction in physical activity observed in our study cohort during COVID-19 had any impact on mother or infant health.

Our study showed that the increase in sedentary behavior and the decrease in physical activity after the pandemic outbreak were still present during 2022, despite that the Swedish restrictions were lifted on 9 February 2022. This finding suggests that pandemic-related obstacles to healthy activity remained, even as other behaviors normalized. Possible explanations include shifts in occupational conditions such as working from home^26^, and psychosocial factors such as anxiety and isolation^27^. Working from home might result in an increase in sedentary behavior as physical activity otherwise would have been accumulated through commuting^28^. This is supported by a Swedish study reporting that hybrid office workers spend more time sitting while working from home, compared with working at the office^29^. Additionally, according to an Irish study, pregnant women experienced increased anxiety, depression, and symptoms of obsessive-compulsive disorder during the COVID-19-era^30^.

The public health interventions to limit the transmission of SARS-CoV-2 virus differed between countries, and the restrictive policies in Sweden were less invasive compared with most other European countries^12^. Sweden had no general lockdown; instead, the strategy focused on mitigating the pandemic. Social distancing was mandatory in certain places (such as public events, bars and restaurants), and strongly recommended elsewhere. Those who had the possibility to work from home were urged to do so^19^. Possibly, pregnant women were more prone to work from home due to fear of contracting COVID-19. They might also have been signed off work earlier to reduce the risk of becoming infected. Given the restrictive policies in Sweden were less invasive compared with many other countries, changes in both sedentary behavior and physical activity levels may have been even more pronounced among pregnant women in countries with lockdown measures. In a future pandemic or a comparable situation, the recommended mitigating strategies might be similar to those used in Sweden during COVID-19, why it is important that the results of this study are accessible to other nations.

A major strength of the study was that accelerometers were used to measure sedentary behavior and physical activity levels objectively^31^. Accelerometers have previously been used to assess physical activity in pregnant women^32^. Moreover, the Actiwatch 2 has been validated against a waist-worn reference physical activity monitor (Actigraph GT3X) and oxygen consumption (metabolic equivalent) with adequate correlations^21^. Another strength was that the length of the activity measurement period (mean 6.7 days) enabled collection of both weekday and weekend activity patterns. This study has also several limitations. Given the observational study design, unmeasured confounders could have influenced study findings. Furthermore, it was only able to evaluate associations, not causality. The generalizability of our results is limited due to local variations in COVID-19 restrictions and societal norms. A further limitation was that we only had data until 2022, and we could therefore not evaluate whether the increase in sedentary behavior and decrease in physical activity remained for a longer period of time. Moreover, light activity was not included in our analyses due to low sensitivity and specificity of the Actiwatch to characterize it^21^. The specific levels of moderate and vigorous activity should also be interpreted with some caution, since the cut-off points suggested by Kemp et al. were determined in a lab setting^21^. We acknowledge that further research is needed to validate the ability of the Actiwatch to measure waking movement behavior in free-living settings.

## Conclusions

The results of this study demonstrated a modest increase in sedentary behavior as well as a small decrease in physical activity levels among pregnant women in Sweden after the outbreak of the COVID-19 pandemic. Moreover, the change in activity patterns remained the following two years. Possible health consequences for pregnant women due to these changes in activity patterns are unknown. Nonetheless, it is important that health providers are aware of the potential negative alterations in activity patterns in the pregnant population following a pandemic or a similar situation. Pregnant women should be encouraged to meet the physical activity guidelines, also during such circumstances, to improve maternal and infant health. As a suggestion for future research, it would be of interest to iterate measurements of sedentary behavior and physical activity levels in pregnant women to evaluate whether the changes observed after the COVID-19 pandemic outbreak persist even after year 2022.

## Data Availability

The datasets generated during and/or analyzed during the current study are available from the corresponding author on reasonable request.

## Abbreviations

β: B coefficient
BMI: Body mass index
CI: Confidence interval
COVID-19: Coronavirus disease 2019
CPM: Counts per minute
SD: Standard deviation
